# The MHC class I *MICA* gene is a histocompatibility antigen in kidney transplantation

**DOI:** 10.1038/s41591-022-01725-2

**Published:** 2022-03-14

**Authors:** Raphael Carapito, Ismail Aouadi, Martin Verniquet, Meiggie Untrau, Angélique Pichot, Thomas Beaudrey, Xavier Bassand, Sébastien Meyer, Loic Faucher, Juliane Posson, Aurore Morlon, Irina Kotova, Florent Delbos, Alexandre Walencik, Alice Aarnink, Anne Kennel, Caroline Suberbielle, Jean-Luc Taupin, Benedict M. Matern, Eric Spierings, Nicolas Congy-Jolivet, Arnaud Essaydi, Peggy Perrin, Antoine Blancher, Dominique Charron, Nezih Cereb, Myriam Maumy-Bertrand, Frédéric Bertrand, Valérie Garrigue, Vincent Pernin, Laurent Weekers, Maarten Naesens, Nassim Kamar, Christophe Legendre, Denis Glotz, Sophie Caillard, Marc Ladrière, Magali Giral, Dany Anglicheau, Caner Süsal, Seiamak Bahram

**Affiliations:** 1 Laboratoire d’ImmunoRhumatologie Moléculaire, Institut National de la Santé et de la Recherche Médicale (INSERM) UMR_S1109, Plateforme GENOMAX, Faculté de Médecine, Fédération Hospitalo-Universitaire OMICARE, Centre de Recherche d’Immunologie et d’Hématologie, Centre de Recherche en Biomédecine de Strasbourg (CRBS), Fédération de Médecine Translationnelle de Strasbourg (FMTS), Université de Strasbourg, Strasbourg, France; 2 Laboratoire d’Excellence (LabEx) TRANSPLANTEX, Faculté de Médecine, Université de Strasbourg, Strasbourg, France; 3 Institut National de la Santé et de la Recherche Médicale (INSERM) Franco (Strasbourg)–Japanese (Nagano) Nextgen HLA Laboratory, Strasbourg, France; 4 Laboratoire d’Immunologie, Plateau Technique de Biologie, Pôle de Biologie, Nouvel Hôpital Civil, Strasbourg, France; 5 Institut Thématique Interdisciplinaire (ITI) de Médecine de Précision de Strasbourg, Strasbourg, France; 6 Nephrology-Transplantation Department, University Hospital, Strasbourg, France; 7 CHU Nantes, Université de Nantes, INSERM, Centre de Recherche en Transplantation et Immunologie, UMR 1064, ITUN, Nantes, France; 8 Paris Translational Research Center for Organ Transplantation, Institut National de la Santé et de la Recherche Médicale (INSERM), UMR_S 970, Paris, France; 9 Kidney Transplant Department, Saint-Louis Hospital, Assistance Publique – Hôpitaux de Paris, Paris, France; 10 BIOMICA SAS, Strasbourg, France; 11 Etablissement Français du Sang (EFS) Centre Pays de la Loire, Laboratoire HLA, Nantes, France; 12 Laboratory of Histocompatibility, Centre Hospitalier Régional Universitaire, Nancy, France; 13 Laboratoire Jean Dausset, Laboratoire d’Immunologie et d’Histocompatibilité, Institut National de la Santé et de la Recherche Médicale (INSERM) UMR_S 976, Human Immunology, Pathophysiology, Immunotherapy (HIPI), Institut de Recherche Saint-Louis Université de Paris, Hôpital Saint-Louis, Paris, France; 14 Center of Translational Immunology, HLA and Tissue Typing, University Medical Center Utrecht, Utrecht, The Netherlands; 15 Laboratoire d’Immunogénétique Moléculaire (LIMT, EA 3034), Faculté de Médecine Purpan, Université Toulouse III (Université Paul Sabatier, UPS), Toulouse, France; 16 Laboratoire d’Immunologie, Centre Hospitalier Universitaire de Toulouse, Toulouse, France; 17 Etablissement Français du Sang (EFS) Grand-Est, Laboratoire HLA, Strasbourg, France; 18 Histogenetics, Ossining, NY USA; 19 Institut de Recherche Mathématique Avancée (IRMA), Centre National de la Recherche Scientifique (CNRS) UMR 7501, Laboratoire d’Excellence (LabEx) Institut de Recherche en Mathématiques, Interactions et Applications (IRMIA), Université de Strasbourg, Strasbourg, France; 20 Service de Néphrologie-Transplantation-Dialyse Péritonéale, Centre Hospitalier Universitaire Lapeyronie, Montpellier, France; 21 Division of Nephrology, University of Liege Hospital (ULiege CHU), Liege, Belgium; 22 Department of Microbiology, Immunology and Transplantation, KU Leuven, Leuven, Belgium; 23 Departments of Nephrology and Organ Transplantation, Centre Hospitalier Universitaire de Rangueil, INSERM UMR1291 - CNRS UMR5051 - Université Toulouse III, Toulouse Institute for Infectious and Inflammatory Diseases (Infinity), Toulouse, Université Toulouse III Paul Sabatier, Toulouse, France; 24 Service de Transplantation Rénale Adulte, Hôpital Necker, Assistance Publique – Hôpitaux de Paris, Université de Paris, Paris, France; 25 Department of Renal Transplantation, Centre Hospitalier Régional Universitaire, Nancy, France; 26 Institut National de la Santé et de la Recherche Médicale (INSERM), UMR_S 1151, Paris, France; 27 Institute of Immunology, Heidelberg University Hospital, Heidelberg, Germany; 28 Transplant Immunology Research Center of Excellence, Koç University, Istanbul, Turkey

**Keywords:** Translational research, Transplant immunology

## Abstract

The identity of histocompatibility loci, besides human leukocyte antigen (*HLA*), remains elusive. The major histocompatibility complex (MHC) class I *MICA* gene is a candidate histocompatibility locus. Here, we investigate its role in a French multicenter cohort of 1,356 kidney transplants. *MICA* mismatches were associated with decreased graft survival (hazard ratio (HR), 2.12; 95% confidence interval (CI): 1.45–3.11; *P* < 0.001). Both before and after transplantation anti-MICA donor-specific antibodies (DSA) were strongly associated with increased antibody-mediated rejection (ABMR) (HR, 3.79; 95% CI: 1.94–7.39; *P* < 0.001; HR, 9.92; 95% CI: 7.43–13.20; *P* < 0.001, respectively). This effect was synergetic with that of anti-HLA DSA before and after transplantation (HR, 25.68; 95% CI: 3.31–199.41; *P* = 0.002; HR, 82.67; 95% CI: 33.67–202.97; *P* < 0.001, respectively). De novo-developed anti-MICA DSA were the most harmful because they were also associated with reduced graft survival (HR, 1.29; 95% CI: 1.05–1.58; *P* = 0.014). Finally, the damaging effect of anti-MICA DSA on graft survival was confirmed in an independent cohort of 168 patients with ABMR (HR, 1.71; 95% CI: 1.02–2.86; *P* = 0.041). In conclusion, assessment of *MICA* matching and immunization for the identification of patients at high risk for transplant rejection and loss is warranted.

## Abstract (web summary)

Analysis of a multicenter cohort of kidney transplants shows that mismatches in the MICA locus and the presence of anti-MICA donor-specific antibodies are associated with reduced graft survival and increased rejection.

## Main

Kidney transplantation is the only curative treatment for end-stage renal disease^1^. The fact that the first successful kidney transplantation in man was between identical twins^2^, along with seminal work in animal models, hinted strongly that a single genetic locus does not govern the clinical outcome of a transplantation, no matter how relevant (such as the major histocompatibility complex (MHC), human leukocyte antigen (HLA)). Indeed, George Snell, in his landmark 1948 study^3^ (as well as subsequent work by himself, and others), identified several dozen histocompatibility loci in the mouse^4^, although close to none has been identified to date in any species (including man).

Fast forward to today, and, owing to the development and refinement of country- and continent-wide allocation processes, perioperative handling of the graft and patients, and selective immunosuppressive drugs that improve transplantation survival mainly by alleviating acute T cell-mediated rejection (TCMR), the number of kidney transplantations is continuously increasing worldwide. However, antibody-mediated rejection (ABMR) is recognized as a major cause of late transplantation failure, and its treatment remains challenging^5^. In addition to the histological findings, a key feature of ABMR is the presence of donor-specific anti-HLA antibodies (DSA)^6^. Nonetheless, in routine clinical care, cases meeting the histological criteria for ABMR but without detectable anti-HLA DSA could represent more than 50% of rejection events^7^. These cases might be explained by the presence of pathogenic antibodies that are produced against other, non-HLA, histocompatibility antigens^8^.

*MHC class I chain-related gene A* (*MICA*; GenBank accession: NM_001177519), discovered almost 30 years ago^9^, encodes a polymorphic non-conventional MHC-encoded class I molecule^10^. The *MICA* gene is located, within the HLA complex, 46 kb centromeric to the *HLA-B* locus^9^. Close to 400 *MICA* alleles have been reported to date^10^. The MICA glycoprotein (Uniprot accession: Q96QC4) is expressed on a restricted number of cell types, mainly epithelial and endothelial cells. MICA binds NKG2D, an activating receptor present on the surface of cytotoxic CD8^+^ αβ and γδ T lymphocytes as well as certain natural killer (NK) cells^10^.

Fifteen years ago Zou et al.^11^ reported the first comprehensive study of the potential involvement of MICA in kidney transplant outcomes. That work, however, was focused only on anti-MICA antibodies and had no information on donor and recipient *MICA* (mis)matching, a situation that has persisted to date given that no study has analyzed simultaneously the sequence-based molecular *MICA* matching and the status of both anti-HLA and anti-MICA DSA in a large cohort for which information about all other relevant covariates was available and included in the final analysis (for review see refs. ^12,13^).

Here, we evaluate the role of *MICA* matching and donor-specific MICA immunization in a retrospective multicenter French cohort of 1,356 patients who had undergone kidney transplantation. All known covariates relevant to graft failure and acute rejection were considered in the analysis. The results highlight the relevance of both *MICA* matching and donor-specific immunization for kidney transplantation outcomes.

## Results

### Baseline characteristics of kidney transplant recipients

The main analysis involved 1,356 patients who underwent kidney transplantation in six French medical centers between 2002 and 2011: 104 in Montpellier, 107 in Paris-Saint-Louis, 188 in Toulouse, 262 in Paris-Necker, 304 in Nancy and 391 in Nantes. The demographics of this study population are listed in Table 1. Most patients were recipients of their first transplant (95%). One hundred and two patients received organs from living donors and 9% of patients received simultaneous kidney–pancreas transplantations. All but two of the relevant covariates for the clinical outcomes analyzed were equally distributed in the *MICA*-matched and -mismatched patients. There were more retransplantations in the *MICA*-matched than in the *MICA*-mismatched groups (10% versus 5%, *P* = 0.04), and *MICA*-mismatched transplantations had more HLA mismatches (*P* < 0.001, *P* < 0.001 and *P* = 0.01 for *HLA-A*, *-B* and *-DRB1* mismatches, respectively; Table 1); both observations are probably due to linkage disequilibrium between *MICA* and *HLA-B*.

**Table 1:** Demographics of the study population by *MICA* matching status

Characteristics	All patients	*MICA* matched	*MICA* mismatched	*P*-value
	(*n* = 1,356)	(*n* = 113)	(*n* = 1,243)	
French transplantation centers^a^, *n* (%)				0.36
Montpellier	104 (8)	6 (5)	98 (8)	
Nancy	304 (22)	20 (18)	284 (23)	
Nantes	391 (29)	38 (34)	353 (28)	
Paris-Necker	262 (19)	27 (24)	235 (19)	
Paris-Saint-Louis	107 (8)	6 (5)	101 (8)	
Toulouse	188 (14)	16 (14)	172 (14)	
Donors				
Age, *n* (%)				0.52
<42 years	364 (27)	29 (26)	335 (27)	
42–63 years	655 (48)	60 (53)	595 (48)	
≥64 years	337 (25)	24 (21)	313 (25)	
Sex, *n* (%)				0.83
Female	571 (42)	46 (41)	525 (42)	
Male	785 (58)	67 (59)	718 (58)	
Living/Deceased donor status, *n* (%)				0.14
Living	102 (8)	13 (11)	89 (7)	
Deceased	1,254 (92)	100 (89)	1,154 (93)	
Recipients				
Age, *n* (%)				0.31
<42 years	348 (26)	35 (31)	313 (25)	
42–61 years	697 (51)	51 (45)	646 (52)	
≥62 years	311 (23)	27 (24)	284 (23)	
Sex, *n* (%)				0.46
Female	432 (32)	32 (28)	400 (32)	
Male	924 (68)	81 (72)	843 (68)	
Median body mass index, *n* (%)				0.19
≤24 kg m^−2^	675 (50)	64 (57)	611 (49)	
>24 kg m^−2^	668 (49)	49 (43)	619 (50)	
Missing	13 (1)	0 (0)	13 (1)	
End-stage kidney disease, *n* (%)				0.65
Potential recurrent nephropathy^b^	79 (6)	5 (4)	74 (6)	
Other	1,277 (94)	108 (96)	1,169 (94)	
Transplantation				
Year of transplantation, *n* (%)				0.66
<2007	488 (36)	38 (33.6)	450 (36.2)	
2007 or after	868 (64)	75 (66.4)	793 (63.8)	
Graft rank, *n* (%)				0.04
First transplant	1,285 (95)	102 (90)	1,183 (95)	
Retransplantation	71 (5)	11 (10)	60 (5)	
Type of transplantation, *n* (%)				0.34
Kidney	1,239 (91)	100 (89)	1,139 (92)	
Kidney and pancreas	117 (9)	13 (11)	104 (8)	
Time from dialysis to transplantation, *n* (%)				0.08
≤27 months	592 (44)	56 (50)	536 (43)	
>27 months	595 (44)	39 (34)	556 (45)	
Missing	169 (12)	18 (16)	151 (12)	
Cold ischemia time, *n* (%)				0.05
≤1,440 min	1,049 (77)	95 (84)	954 (77)	
>1,440 min	298 (22)	16 (14)	282 (23)	
Delayed graft function, *n* (%)				0.24
No	854 (63)	80 (71)	774 (62)	
Yes	391 (29)	28 (25)	363 (29)	
Missing	111 (8)	5 (4)	106 (9)	
Donor–Recipient CMV status, *n* (%)				0.83
Negative–Negative	277 (20)	22 (19)	255 (20)	
Negative–Positive	362 (27)	29 (26)	333 (27)	
Positive–Negative	245 (18)	24 (21)	221 (18)	
Positive–Positive	463 (34)	37 (33)	426 (34)	
Missing	9 (1)	1 (1)	8 (1)	
Induction treatment^c^, *n* (%)				0.36
Non-depleting induction	637 (47)	52 (46)	585 (47)	
Depleting induction	558 (41)	43 (38)	515 (41)	
No induction treatment	161 (12)	18 (16)	143 (12)	
Immunologic characteristics at time of transplantation				
HLA-A mismatches, *n* (%)				<0.001^e^
0	256 (19)	37 (33)	219 (18)	
1 or 2	1,100 (81)	76 (67)	1,024 (82)	
HLA-B mismatches, *n* (%)				<0.001^f^
0	134 (10)	53 (47)	81 (7)	
1 or 2	1,222 (90)	60 (53)	1,162 (93)	
HLA-DRB1 mismatches, *n* (%)				0.01
0	320 (24)	38 (34)	282 (23)	
1 or 2	1,036 (76)	75 (66)	961 (77)	
Anti-HLA class I antibodies^d^, *n* (%)				0.25
No	1,253 (92.4)	108 (95.6)	1,145 (92.1)	
Yes	103 (7.6)	105 (4.4)	98 (7.9)	
Anti-HLA class II antibodies^d^, *n* (%)				
No	1,257 (92.7)	107 (94.7)	1,150 (92.5)	0.53
Yes	98 (7.2)	6 (5.3)	92 (7.4)	
Missing	1 (0.1)	0 (0)	1 (0.1)	
Anti-HLA DSA antibodies before transplantation^d^, *n* (%)			1	
No	1,294 (95)	108 (96)	1,186 (95)	
Yes	62 (5)	5 (4)	57 (5)	

CMV, cytomegalovirus.

All clinical variables of the table were used for adjustment in the multivariate models.

Two-sided *P* values were determined using the Pearson’s chi-squared test or the Fisher’s exact test and were not corrected for multiple testing. Exact *P* values: ^e^1.40 × 10^−4^, ^f^2.20 × 10^−16^.

^a^Patients received their transplants at six centers that were members of the DIVAT (‘Données Informatisées et VAlidées en Transplantation’) consortium.

^b^Potential recurrent nephropathy includes: focal segmental glomerulosclerosis, IgA nephropathy, type I and II membranoproliferative glomerulonephritis, membranous glomerulonephritis, granulomatosis with polyangiitis, systemic lupus erythematosus, scleroderma and hemolytic uremic syndrome.

^c^Induction therapy was performed with anti-thymocyte globulin or anti-CD3 antibody (depleting) or anti-interleukin 2 receptor antibody (non-depleting).

^d^Pre-transplantation anti-HLA immunization was determined by complement-dependent cytotoxicity, ELISA or Luminex.

### *MICA* matching and graft survival

**Fig. 1:**
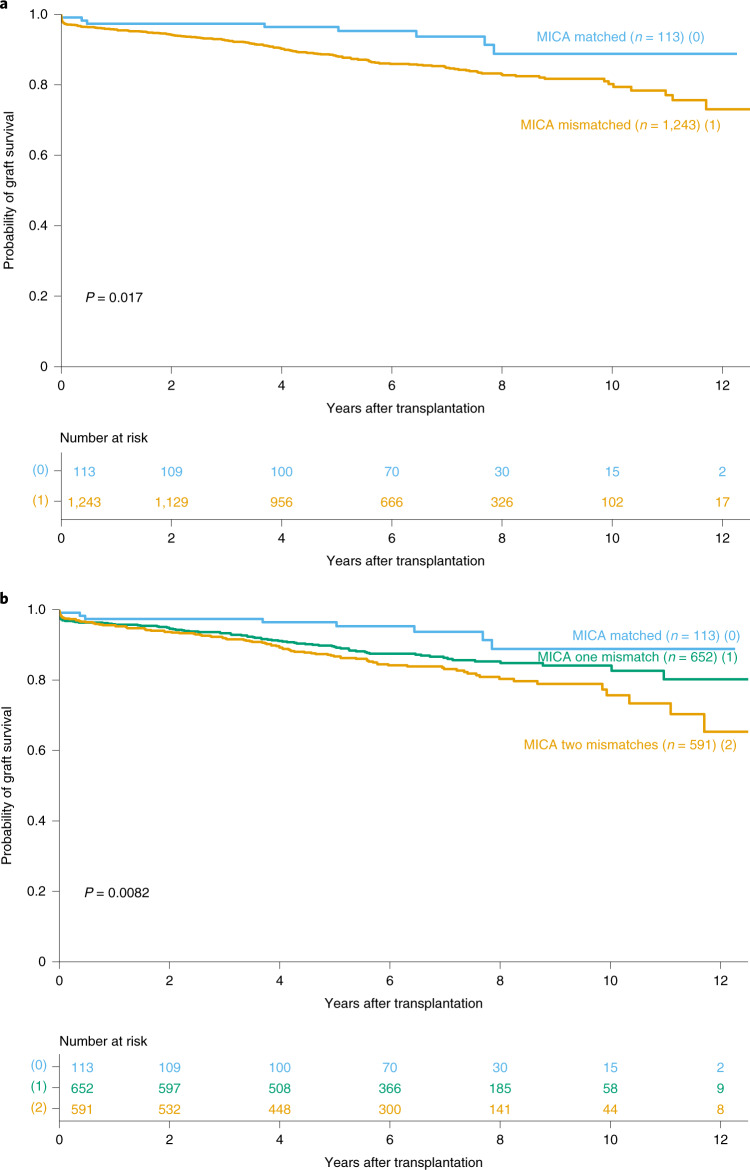
Kaplan–Meier curves for kidney graft survival according to *MICA* matching status. The probability of graft survival is shown for matched versus mismatched patients using the presence or absence of mismatches at the *MICA* locus (**a**) or the number of mismatches (**b**) as classification criteria. *P* values were determined using the two-sided log-rank test without correction. Source data

The median follow-up after transplantation was 6.3 years, with a maximum of 12.9 years. The median follow-up was 6.5 and 6.3 years for the *MICA*-matched and -mismatched patients, respectively. A total of 192 patients (14.2%) had graft failure during follow-up; 1,208 patients (89.1%) survived. Compared with *MICA*-mismatched patients, *MICA*-matched patients had a significantly improved graft survival rate (*P*_log-rank_ = 0.017), which was the primary endpoint of the study (Fig. 1a). At 5 years after transplantation, graft survival was 96% and 88% for *MICA*-matched and -mismatched patients, respectively, and this difference in survival rate was also observed when comparing the different mismatching possibilities at the *MICA* locus (0 versus 1 versus 2 mismatches, *P*_log-rank_ = 0.008) (Fig. 1b). The most important impact on graft survival was observed for the case of two mismatches, with rates of 87% and 76% at 5 and 10 years after transplantation, respectively. Based on multivariate Cox regression, *MICA* mismatching was an independent factor associated with graft loss (HR, 2.12; 95% CI: 1.45–3.11; *P* < 0.001). Other independent risk factors in the model included age of the donor and recipient, dialysis duration, initial nephropathy, older transplantations, delayed graft function and absence of induction treatment (Table 2). *HLA-A*, *-B* and *-DRB1* mismatching at a low level of resolution had no impact on graft failure (Extended Data Table 1).

**Table 2:** Multivariate factors associated with kidney graft loss^a^

Factors	HR (95% CI)	*P* value
Age of donor (≥64 years)	2.36 (1.46–3.81)	<0.001^b^
Age of recipient (≥62 years)	1.47 (1.13–1.91)	0.004
Time from dialysis to transplantation (>27 months)	1.36 (1.06–1.74)	0.016
Potential recurrent nephropathy	1.53 (1.07–2.18)	0.019
Transplantation before 2007	1.27 (1.01–1.61)	0.039
Delayed graft function (≥1 day)	1.36 (1.20–1.55)	<0.001^c^
No induction treatment	1.48 (1.05–2.08)	0.024
1 or 2 *MICA* mismatches	2.12 (1.45–3.11)	<0.001^d^

^a^Multivariate Cox regression was carried out using death-censored graft survival and included all covariates listed in Table 1. Two-sided *P* values were calculated using Wald’s test without correction for multiple testing. Exact *P* values: ^b^4.71 × 10^−4^, ^c^1.50 × 10^−6^, ^d^1.18 × 10^−4^.

**Extended Data Fig. 1:**
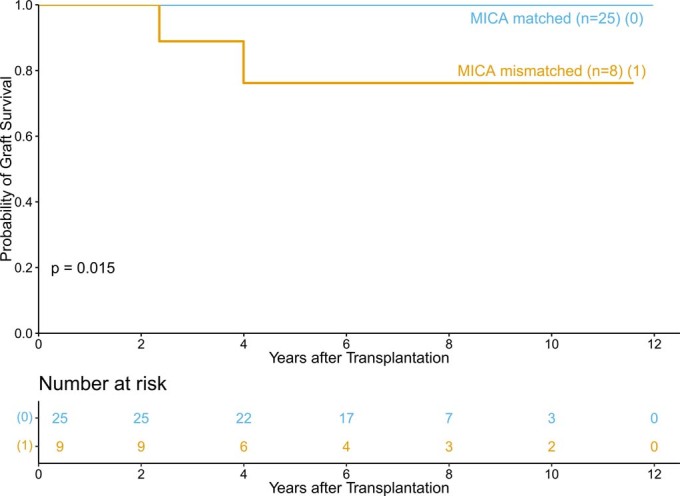
Kaplan–Meier curves for kidney graft survival according to the *MICA* matching status in *HLA-B* matched patients as determined by high-resolution HLA-typing. The probability of graft survival is shown for patients matched versus mismatched at the MICA locus using presence/absence of mismatches. Two-sided log-rank test p-value without correction is shown. Source data

To exclude potential bias due to the difference in the resolution of *MICA* and *HLA* genotypes, we analyzed a subset of 862 transplants in which both donor and recipient were retrospectively *HLA*-typed at second-field resolution, which corresponds to allele-level resolution of *MICA* typing. Multivariate analysis confirmed the *HLA*-independent association of *MICA* mismatches with a higher incidence of graft loss (HR, 1.53; 95% CI: 1.07–2.19; *P* = 0.018; Extended Data Table 2). Other risk factors for graft loss in the model included age of the donor and recipient, dialysis duration, initial nephropathy, pre-transplantation anti-HLA DSA, number of transplantations, absence of induction treatment, depleting induction treatment and *HLA-DQB1* mismatches (Extended Data Table 2). We also confirmed the *HLA-B*-independent effect of *MICA* by analyzing *HLA-B*-matched transplantations in this subset of transplants (*n* = 33), in which *MICA* mismatches were still associated with lower graft survival (*P*_log-rank_ = 0.015, Extended Data Fig. 1).

Finally, *MICA* eplet mismatches had a similar association with graft loss, but did not reach statistical significance (*P*_log-rank_ = 0.11, Supplementary Fig. 1).

### Impact of preformed anti-MICA DSA on graft outcome

**Fig. 2:**
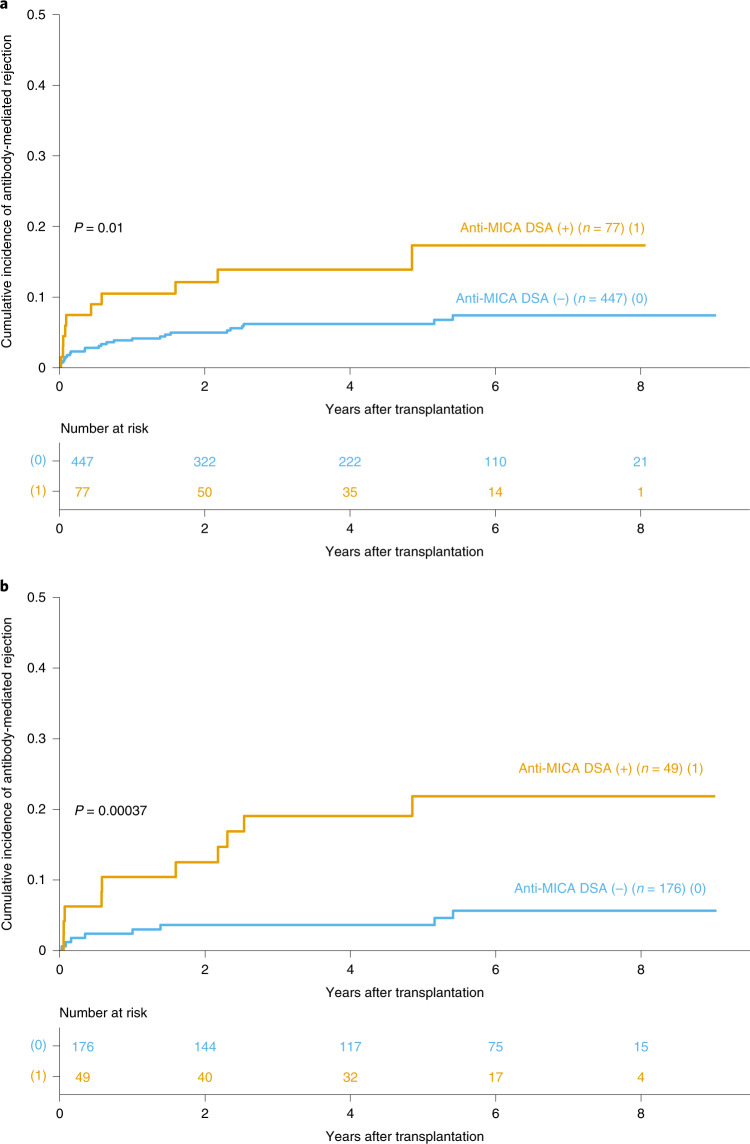
Cumulative incidence of antibody-mediated rejection according to anti-MICA DSA status. The cumulative incidence of antibody-mediated rejection is shown for patients with versus those without preformed anti-MICA DSA (**a**) and for patients with versus those without anti-MICA DSA 1 year after transplantation (**b**). *P* values were determined using the two-sided log-rank test without correction. Source data

Although there is no functional analogy between HLA and MICA molecules, however, to establish whether the observed lower graft survival associated with donor–recipient *MICA* mismatches might be explained by immunization against MICA (similarly to the situation between HLA mismatches and anti-HLA DSA), we analyzed the pre-transplant sera of 524 patients for the presence of anti-MICA DSA. In this subset of patients, the median follow-up was 5.80 years (with a maximum at 9.58 years) in those with anti-MICA DSA, and 6.04 years (with a maximum at 10.09 years) in those without anti-MICA DSA (Supplementary Table 1). Given that acute rejection is a major cause of kidney transplantation failure (HR, 2.64; 95% CI: 2.15–3.25; *P* < 0.001, Extended Data Table 3), we assessed whether donor-specific immunization against MICA had a role in this clinical event, which was the secondary endpoint of the study. Acute clinical rejection developed in 77 patients: TCMR in 52 (9.9%) and ABMR in 35 (6.7%), and of those 10 were mixed-type rejections (1.9%). The presence of anti-MICA DSA was found to be an independent risk factor for acute rejection, with a borderline but significant effect on TCMR (HR, 2.11; 95% CI: 1.01–4.42; *P* = 0.047) and a more important effect on ABMR (HR, 3.79; 95% CI: 1.94–7.39; *P* < 0.001; Fig. 2a and Table 3). Preformed anti-MICA DSA were not associated with graft loss (HR, 1.32; 95% CI: 0.82–2.10; *P* = 0.25; Table 3). The association of eplet-specific anti-MICA DSA with ABMR was similar to that of all anti-MICA DSA (Supplementary Fig. 2 and Extended Data Table 4).

**Table 3:** Impact of pre- and post-transplantation anti-MICA DSA on kidney graft loss and acute rejection^a^

Endpoint	Preformed anti-MICA DSA (*n* = 524)		Post-transplantation (1 year) anti-MICA DSA (*n* = 225)^b^		Post-transplantation (1 year) de novo anti-MICA DSA (n = 225)^b^	
	HR (95% CI)	*P* value	HR (95% CI)	*P* value	HR (95% CI)	P value
Overall graft loss	1.67 (1.28–2.18)	<0.001^c^	1.64 (1.11–2.42)	0.013	0.98 (0.39–2.47)	0.970
Death-censored graft loss	1.32 (0.82–2.10)	0.250	1.02 (0.73–1.42)	0.910	1.29 (1.05–1.58)	0.014
Acute rejection	2.28 (1.40–3.71)	<0.001^d^	1.98 (1.26–3.10)	0.003	1.94 (1.88–2.01)	<0.001^f^
TCMR	2.11 (1.01–4.42)	0.047	1.60 (1.01–2.53)	0.043	1.84 (1.68–2.01)	<0.001^g^
ABMR	3.79 (1.94–7.39)	<0.001^e^	9.92 (7.43–13.20)	< 0.001	3.30 (2.25–4.85)	<0.001^h^

^a^Multivariate Cox regression included all covariates listed in Table 1. Two-sided *P* values were calculated using Wald’s test without correction for multiple testing. Exact *P* values: ^b^The same 225 patients were analyzed for 1 year anti-MICA DSA and for 1 year de novo anti-MICA DSA. ^c^1.32 × 10^−4^, ^d^8.78 × 10^−4^, ^e^9.49 × 10^−5^, ^f^1.57 × 10^−258^, ^g^5.71 × 10^−37^, ^h^2.48 × 10^−9^.

### One year post-transplant anti-MICA DSA and graft outcome

Immunization against MICA was analyzed using 225 serum samples collected 1 year after transplantation. In this subset of patients the median follow-up was 7.37 years (with a maximum at 9.58 years) and 7.34 years (with a maximum at 9.65 years) in those with and without anti-MICA DSA, respectively (Supplementary Table 2).

Although the presence of anti-MICA DSA at 1 year after transplantation was not associated with a higher incidence of graft failure, it was a risk factor for both TCMR (HR, 1.60; 95% CI: 1.01–2.53; *P* = 0.043) and ABMR (HR, 9.92; 95% CI: 7.43–13.20; *P* < 0.001; Fig. 2b and Table 3). Moreover, these associations were maintained when considering only the de novo fraction of these antibodies. Interestingly, the presence of de novo anti-MICA DSA was also a risk factor for graft survival (HR, 1.29; 95% CI: 1.05–1.58; *P* = 0.014; Table 3). Finally, the presence of anti-MICA DSA after transplantation was associated with a higher frequency of *MICA* mismatches whether considering all DSA present at 1 year after transplantation (0% versus 24.6% in matched versus mismatched patients, *P* = 0.0017) or only the de novo fraction of these antibodies (0% versus 13.5% in matched versus mismatched patients, *P* = 0.05).

We also tested whether specific *MICA* alleles were more prone to elicit DSA than others. For this purpose, we conducted a chi-squared test for equality of proportions on the proportion of individuals developing de novo anti-MICA DSA conditional on the presence of a specific *MICA* allele in the donor. There was no specific *MICA* allele that was associated with a higher rate of de novo anti-MICA DSA (Extended Data Table 5). Finally, when considering only eplet-specific anti-MICA DSA, the association with ABMR was similar to that of all anti-MICA DSA (Supplementary Fig. 3 and Extended Data Table 4).

**Fig. 3:**
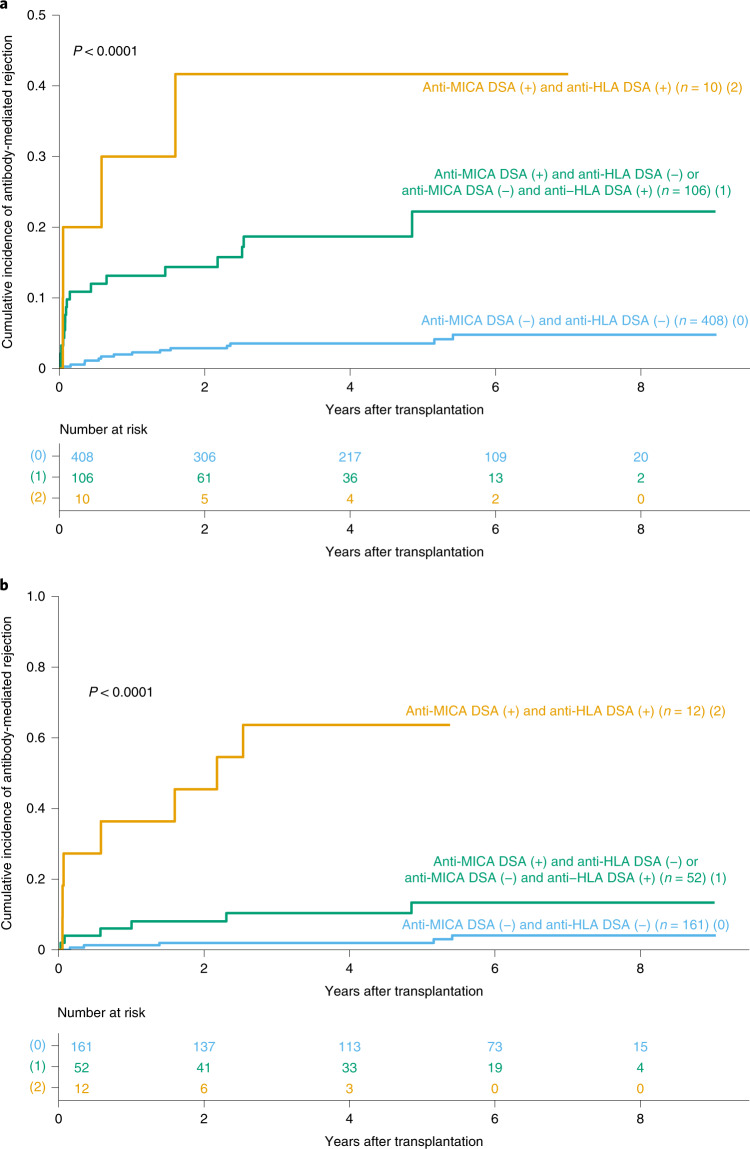
Cumulative incidence of antibody-mediated rejection according to anti-MICA and anti-HLA DSA status. The cumulative incidence of antibody-mediated rejection is shown for patients without DSA, with anti-MICA or anti-HLA DSA, and with both anti-MICA and anti-HLA DSA. The analysis was carried out for preformed (**a**) and post-transplantation DSA (**b**). *P* values were determined using the two-sided log-rank test without correction. Exact *P* values: **a**, *P* = 1.44 × 10^−10^; **b**, *P* = 5.03 × 10^−17^. Source data

### Synergetic effect of anti-MICA and anti-HLA DSA on ABMR

To evaluate the additive or synergetic impact of anti-MICA and anti-HLA DSA on ABMR, we analyzed the cumulative incidence of ABMR as a function of the presence or the absence of these antibodies before and after transplantation, as determined by single-antigen Luminex assays. The presence of anti-MICA or anti-HLA DSA, before and after transplantation, was a risk factor for ABMR (Fig. 3). In addition, both anti-MICA and anti-HLA DSA had an independent effect on ABMR, before and after transplantation (Extended Data Table 6). Interestingly, the risk of developing ABMR was highest when both types of antibodies were present (HR, 25.68; 95% CI: 3.31–199.41; *P* = 0.002 for preformed antibodies and HR, 82.67; 95% CI: 33.67–202.97; *P* < 0.001 for post-transplant antibodies; Fig. 3 and Extended Data Table 6).

### Anti-MICA DSA and graft survival in an independent cohort

**Extended Data Fig. 2:**
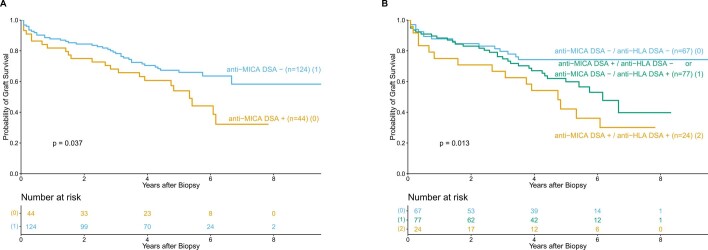
Kaplan–Meier curves for kidney graft survival according to anti-MICA and anti-HLA DSA antibodies in an independent cohort with ABMR. The probability of graft survival is shown for patients with anti-MICA DSA at the time of biopsy versus those without anti-MICA DSA (panel A) and for patients without DSA, with anti-MICA or anti-HLA DSA, and with both anti-MICA and anti-HLA DSA (panel B). Two-sided log-rank test P values without correction are shown. Source data

To further evaluate the role of anti-MICA DSA, we analyzed an independent cohort of 168 patients who had an episode of ABMR with or without anti-HLA DSA between 2013 and 2018. The median follow-up time after biopsy was 4.15 years (with a maximum at 7.90 years) and 4.47 years (with a maximum at 8.18 years) in those without (*n* = 124) and with (*n* = 44) anti-MICA DSA, respectively (Supplementary Table 3). The presence of anti-MICA DSA at the time of the diagnostic biopsy was associated with a decreased graft survival rate (HR, 1.71; 95% CI: 1.02–2.86; *P* = 0.041), as shown by a difference of 19% in survival at 6 years between patients with and without MICA DSA (Extended Data Fig. 2a). Of note, the graft survival was worst when both anti-MICA and anti-HLA DSA antibodies were present, confirming a synergetic effect of these antibodies on graft survival (Extended Data Fig. 2b).

## Discussion

Here, we report that kidney transplantation from *MICA*-mismatched donors carries a significantly higher risk of graft failure. The lower graft survival can be explained by an increased rate of ABMR, which is independently associated with anti-MICA DSA. The present data formally define MICA as a bona fide transplantation antigen in kidney organ transplants and provide the rationale for including *MICA* genotyping and immunization monitoring in the pre- and post-transplantation workup. These results could be contextualized within several key, convergent and divergent, aspects of HLA and *MIC* genetics and immunobiology.

On the genetic side, one of the major challenges in any association study involving MHC genes is the high degree of linkage disequilibrium within the complex, here exemplified using that between *MICA* and *HLA-B*, which are separated by a 46 kb stretch of DNA (Extended Data Table 7 provides an update on linkage disequilibrium between *MICA* and all classical HLA genes). This could mean that some of the observed associations could indeed be due to linkage disequilibrium rather than being a primary association. However, the contribution of linkage disequilibrium to our results was ruled out by inclusion of all HLA mismatches as covariates in the multivariate Cox model, as well as by the observation of a still-significant association of graft survival with *MICA* mismatches in the subset of donors and recipients who were allele-matched for *HLA-B* (Table 2 and Extended Data Fig. 1). This is also in line with an independent assessment of the contribution of *MICA* mismatching to the outcome of hematopoietic cell transplants^14,15^.

Despite attention to long-term follow-up, it should also be noted that *HLA-A*, -*B* and -*DRB1* mismatches had no impact on graft survival in this cohort (Extended Data Tables 1 and 2). This is probably due to the comparatively smaller size of our cohort with respect to large, (multi) continent-wide cohorts, which have been able to show HLA-dependent disease outcome in kidney transplant recipients; for example the Collaborative Transplant Study (CTS), UK Transplant and Eurotransplant, with more than 100,000 donor–recipient pairs^16,17^. The necessity of having large cohorts to show an *HLA*-mismatching effect is due to the following: there is only a 15% survival difference at 10 years after transplantation between fully matched kidneys and kidneys mismatched for both alleles at *HLA-A*, -*B* and *-DRB1* loci^18^; and the magnitude of this effect has decreased over the years as a positive effect from many allocation policies taking matching into account^19^. The absence of *MICA* from these allocation policies may indeed explain why fewer donor–recipient pairs are needed to highlight a significant impact of *MICA* mismatching on graft outcome and, in consequence, to further incentivize its inclusion in a pre-transplant workup. Interestingly, in the subset of transplants with high-resolution typing of six *HLA* loci, only *HLA-DQB1* mismatches were associated with lower graft survival (HR, 1.71; 95% CI: 1.35–2.17; *P* < 0.001; Extended Data Table 2). This observation is in line with recent reports showing associations of *HLA-DQB1* mismatches with acute rejection^20,21^ and decreased graft survival^22^.

On the biological front, despite the fact that both MICA and HLA class I genes and molecules have a similar and unique tri-dimensional structure, major differences exist in their respective functions, for example HLA class I require both the β_2_-microglobulin and an endogenously derived peptide antigen for proper surface expression, and interact with the T cell receptor, whereas MICA does not require either β_2_-microglobulin or any peptide cargo for surface expression and interacts with a distinct receptor, NKG2D. Other differences include (and this is despite the fact that after *HLA* genes, *MIC* genes are the most polymorphic loci in the human genome) a substantially higher degree of diversity (for example, >8,000 *HLA-B* alleles versus >300 *MICA* alleles, vastly higher numbers of polymorphic positions for HLA molecules than MICA; see http://hla.alleles.org/alleles/index.html), and substantially stronger tissue expression for *HLA* class I than *MICA* (see comparative RNA sequencing data at https://gtexportal.org/home/multiGeneQueryPage/MICA,HLA-B). Incidentally, the last two facts are probably the reason for the higher antigenicity of HLA compared with MICA molecules, as evidenced by the disparity in the level of mean fluorescence intensity for anti-MICA compared with anti-HLA antibodies.

Independently of the influence of *MICA* genetic incompatibility on graft outcome, our study equally showed that the presence of pre- and post-transplantation anti-MICA DSA was strongly associated with an increased incidence of ABMR (Fig. 2 and Table 3), an effect that was independent of, and synergetic with, that of anti-HLA DSA (Fig. 3 and Extended Data Table 1). Indeed, because they were also associated with transplantation failure, de novo anti-MICA DSA appeared to be more harmful than preformed antibodies (Table 3). Given that these harmful antibodies are associated with *MICA* mismatches (0% versus 13.5% of patients with de novo antibodies in *MICA*-matched and -mismatched transplantations, respectively), they can be anticipated by performing pre-transplant *MICA* genotyping. Finally, anti-MICA DSA were confirmed to be harmful because they were associated with graft loss in an independent cohort of ABMR patients (Extended Data Fig. 2). Some of these observations were made in two subcohorts (pre-transplant and post-transplant) of the initial (master) cohort. Of note, patient inclusion in each subcohort depended solely on the availability of their sera (Supplementary Tables 1 and 2); and the incidence of the main endpoint analyzed in these subcohorts, ABMR, was not significantly different from that observed in the main cohort, that is: 6.3% in the main cohort versus 6.7% in the pre-transplant cohort (95% CI: 4.6–7.8; *P* = 0.57) and 6.3% versus 8.1% in the post-transplant subcohort (95% CI: 3.6–9.4; *P* = 0.17). Importantly, when analyzing the demographics and distribution of covariates in these two subcohorts, similarly to what had been already observed in the main cohort between *MICA*-matched and -mismatched transplantations, there were more retransplantations in the group with anti-MICA DSA than in the group without anti-MICA DSA (pre-transplant subcohort: 15.6% versus 5.8%, *P* = 0.005, Supplementary Table 1, and post-transplant subcohort: 14.3% versus 4%, *P* = 0.02, Supplementary Table 2). This observation could be explained by the fact that patients who had more than one transplantation are generally more immunized. The other unique covariate that was not equally distributed in patients with and without anti-MICA DSA was the proportion of potential recurrent nephropathies (11.7% versus 4.7%, *P* = 0.03), which was probably due to the fact that there were more retransplantations in these patients with potentially recurrent nephropathies than in those without (13.3% versus 6.9%).

Based on structural accessibility, MICA polymorphic residues can be grouped in small patches of surface-exposed amino acids, called eplets, using HLAMmatchmaker^23^. According to work by Duquesnoy et al., first for classical HLA molecules^24^ and later for MICA^25^, donor-specific eplets are thought to represent surface-accessible polymorphic amino acids prone to elicit DSA. Even though this theory has been verified for HLA (for example ref. ^26^), when considering *MICA* eplet mismatches instead of global *MICA* mismatches and eplet-specific anti-MICA DSA instead of all donor-specific anti-MICA DSA, similar results but no improvements in terms of associations with graft loss or ABMR could be evidenced in our dataset (Supplementary Figs. 1–3 and Extended Data Table 4). This discrepancy with HLA might be explained by the fact that *MICA*-mismatched alleles considered as matched at the eplet level may have immunogenic characteristics that cannot be identified using the HLAMmatchmaker approach. The limited number of reported eplet validation sera for MICA and the less extensive knowledge of MICA structures and polymorphisms may also be reasons for the non-superiority of associations measured when restricting the analysis to eplets. To sum up, in contrast to the HLA setting, the global and eplet mismatching models performed equally well for MICA. Although immunologically more correct, the eplet model and the number of identified eplets for MICA might still need improvements to demonstrate its superiority over the global mismatching model. The outcomes of this study warrant further detailed investigations on the eplet model for MICA.

In conclusion, molecular typing of *MICA* in association with screening for anti-MICA antibodies has the potential to lower the incidence of kidney transplantation rejection and loss.

## Methods

### Study design and oversight

The aim of this retrospective histocompatibility study was to examine whether donor–recipient matching at the *MICA* locus improves the outcomes of kidney transplantation. Kidney transplant recipients (and their donors) from seven French centers (Montpellier, Paris–Saint-Louis, Toulouse, Paris–Necker, Nancy, Nantes and Strasbourg) were enrolled. Genomic DNA and sera were collected in each participating center in the course of routine medical care and histocompatibility geno- and serotyping. The study was approved by the institutional review boards (IRBs) of Nantes University Hospital (CPP Grand Ouest DC-2011-1399, on behalf of all participating centers, except Strasbourg) and Strasbourg University Hospital (CPP Est number DC-2013-1990). The study was performed according to the principles of the Helsinki declaration. Written informed consent was obtained from all participants of both the initial and the independent cohorts.

### Patients and donors

The study population consisted of 1,356 kidney transplant recipients (and donors) from six of the seven centers (Montpellier, Paris–Saint-Louis, Toulouse, Paris–Necker, Nancy and Nantes) who underwent kidney transplantation between 2002 and 2011. The patients who survived and were not lost to follow-up during the study were followed until 1 January 2015. All patients who underwent transplantation and died during the study period were included in the analysis. The transplantation allocation rules were the same for all seven centers and followed the recommendations of the French national agency for organ procurement (Agence de la biomédecine, Paris, France). All transplants were ABO compatible, and cross-matching for immunoglobulin (Ig)G T cell and B cell complement-dependent cytotoxicity was negative for all patients before transplantation. An independent cohort of 168 patients from Strasbourg University Hospital with a biopsy-proven acute ABMR episode that occurred between 2013 and 2018 was also analyzed. These patients had ABMR-specific lesions with (*n* = 81) or without (*n* = 87) anti-HLA DSA.

### *MICA* and *HLA* genotyping

Genotyping of *MICA* in all donors and recipients was carried out using sequence-based typing: exons 2, 3 and 4 were bidirectionally Sanger-sequenced, and the transmembrane microsatellite polymorphism was genotyped as follows. A fragment spanning exons 2–5 of the *MICA* gene was amplified using polymerase chain reaction (PCR) on genomic DNA with a forward (5'-CGTTCTTGTCCCTTTGCCCGTGTGC-3') and a reverse (5'-GATGCTGCCCCCATTCCCTTCCCAA-3') primer using the Expand Long Template PCR System (Roche), following the manufacturer’s recommendations. After purification with the QIAquick PCR Purification Kit (QIAGEN), the PCR product was directly sequenced with the BigDye Terminator v3.1 Cycle sequencing kit and run on a 96 capillary ABI3730XL Genetic Analyzer (ThermoFisher Scientific). Sequences were analyzed using Seqscape v2.6 (ThermoFisher Scientific). The MICA-transmembrane (TM) coding region was amplified with a forward primer labeled at the 5' end with 6-carboxyfluorescein (FAM) (5'-CCTTTTTTTCAGGGAAAGTGC-3') and a reverse primer (5'-CCTTACCATCTCCAGAAACTGC-3'), using GoTaq Polymerase (Promega) following the manufacturer’s instructions. To determine the number of triplet repeats in the TM region of the *MICA* gene, PCR products were run on a 96 capillary ABI3130xl Genetic Analyzer and their sizes were determined using Genemapper v4.0 (ThermoFisher Scientific). MICA-TM genotypes (MICA A4, A5, A5.1, A6 or A9) were determined by comparing the sizes of the obtained fragments with controls of known genotypes^27^. Final MICA genotypes were assigned using an in-house developed VBA code (Microsoft Excel) compiling sequence data and MICA-TM genotypes. Finally, ambiguous results were resolved by PCR amplification with sequence-specific primers. Upon completion of this procedure, analysis of matching and mismatching between donors and recipients was performed at allele-level resolution (second field in the HLA nomenclature^28^). *HLA* genotyping data were retrieved from participating centers, with a first-field resolution for *HLA-A*, -*B* and *-DRB1* loci. Retrospective second-field-resolution *HLA-A*, *-B*, *-C*, *-DRB1*, *-DQB1* and *-DPB1* genotyping was performed by sequence-based typing on a subset of 862 donor–recipient pairs for whom sufficient DNA was available.

### Anti-HLA and -MICA antibody testing

In the main cohort we used 524 pre-transplant serum samples and 225 post-transplant (at 1 year) serum samples to evaluate levels of anti-HLA and MICA DSA with the respective LABScreen Single Antigen kits (One Lambda) following the manufacturer’s instructions. The same kits and conditions were used to evaluate anti-HLA and anti-MICA DSA in an independent cohort of 168 patients who had an episode of ABMR at the time of diagnostic biopsy. Antibodies were detected based on the mean fluorescence intensity (MFI) for each bead coated with an HLA or MICA antigen, as normalized to the value measured with the negative control serum using the baseline method. All beads with normalized MFI higher than 500 or 100 were considered positive for HLA and MICA, respectively. The MFI cut-off for positivity of anti-MICA DSA was chosen based on a receiver operating characteristic analysis (Supplementary Fig. 4). The maximum MFI of DSA was defined as the highest ranked donor-specific bead. For the remaining patients, anti-HLA antibody testing was performed using either complement-dependent cytotoxicity, ELISA or Luminex-based tests.

### Statistical analyses

The primary endpoint of the study was the post-transplantation time to graft failure, which was censored at the time of the last follow-up or death. The secondary endpoint was the first episode of acute rejection. All acute rejection episodes were biopsy proven and classified according to the Banff classification^29^. Acute rejection episodes were classified into acute TCMR and ABMR. Delayed graft function was defined as the use of dialysis within 7 days after transplantation, except in the case of one-off dialysis for hyperkalemia or fluid overload, which was not counted as delayed graft function. All statistical models were adjusted for the center effect^30^ and included the following covariates: donor age, recipient age, donor sex, recipient sex, deceased–living status of donor, recipient body mass index, cause of end-stage kidney disease, year of transplantation, graft rank, type of transplantation, time from dialysis to transplantation, cold ischemia time, delayed graft function, donor and recipient cytomegalovirus status, induction treatment, HLA mismatches, and pre-transplantation anti-HLA class I and II antibodies including those that were donor-specific. Continuous variables were transformed into categorical variables. We used counts and percentages to describe variables. A chi-squared test for independence (or Fisher’s exact test if appropriate) was used to examine the association between the *MICA* matching variable and each other variable.

Probabilities of graft survival and univariate analysis were assessed using Kaplan–Meier curves and the log-rank test. Cox proportional hazards models were applied to quantify hazard ratios and 95% confidence intervals. The association of factors with graft survival and acute rejection was determined by multivariate Cox regression analysis. Multivariate models were all adjusted for center effects, and all models were evaluated for proportional hazards assumptions. All reported *P* values were two-sided and were considered to indicate statistical significance if less than 0.05. Statistical analysis was performed using the computing environment R (v4.0.2) with the CRAN survival package (https://cran.rproject.org/web/packages/survival/index.html).

### Reporting Summary

Further information on research design is available in the Nature Research Reporting Summary linked to this article.

## Online content

Any methods, additional references, Nature Research reporting summaries, source data, extended data, supplementary information, acknowledgements, peer review information; details of author contributions and competing interests; and statements of data and code availability are available at 10.1038/s41591-022-01725-2.

### Supplementary information

Supplementary Information Supplementary Figs. 1–4, Supplementary Tables 1–3.

Reporting Summary

### Source data

Source Data Fig. 1 Statistical source data for graft survival curves.

Source Data Fig. 2 Statistical source data for cumulative rejection incidence curves.

Source Data Fig. 3 Statistical source data for graft survival curves.

Source Data Extended Data Fig. 1 Statistical source data for graft survival curves.

Source Data Extended Data Fig. 2 Statistical source data for graft survival curves.

## Figures and Tables

**Extended Data Table 1:**
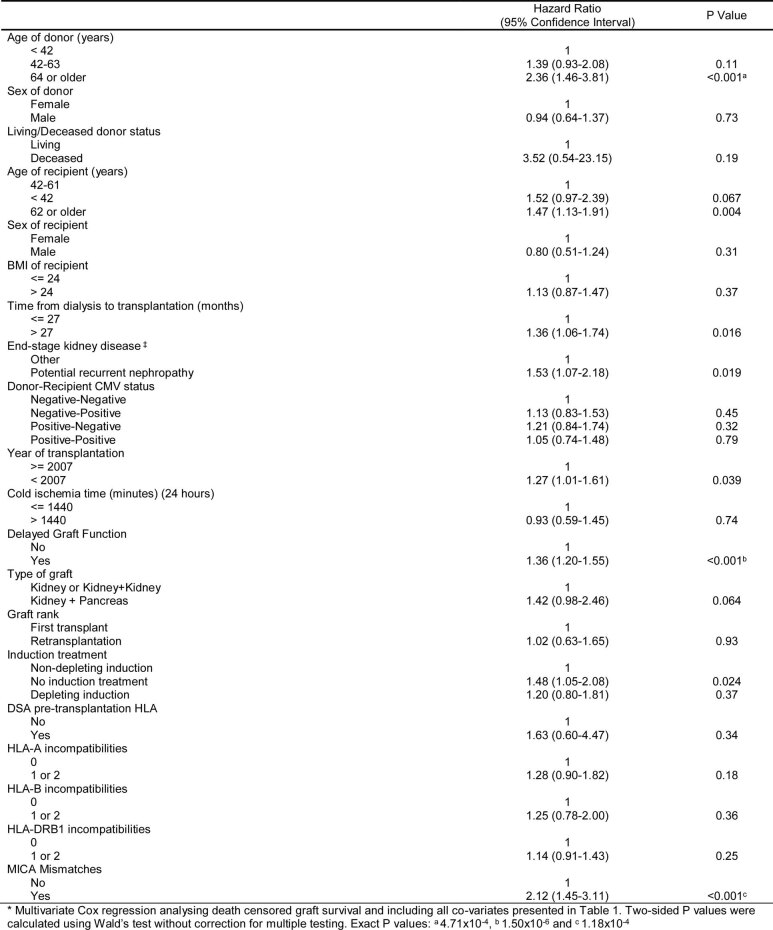
Multivariate Cox regression of possible factors associated with kidney graft loss*

**Extended Data Table 2:**
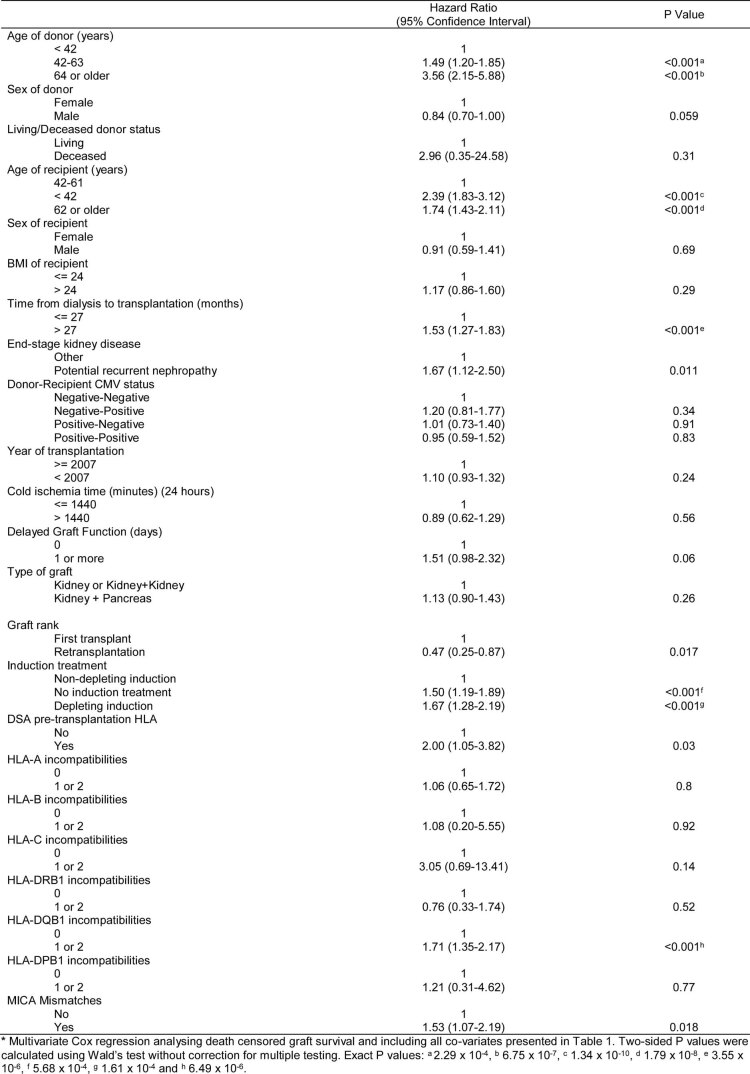
Multivariate Cox regression of possible factors associated with kidney graft loss in a subset of 862 transplants with high-resolution HLA-typing*

**Extended Data Table 3:**
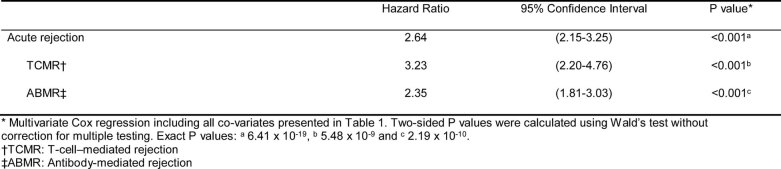
Analysis of the impact of acute rejection on allograft survival by multivariate analysis*

**Extended Data Table 4:**
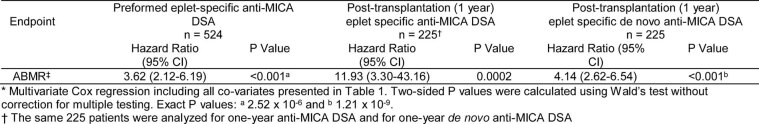
Impact of pre and post-transplantation eplet-specific anti-MICA DSA on antibody-mediated rejection*

**Extended Data Table 5:**
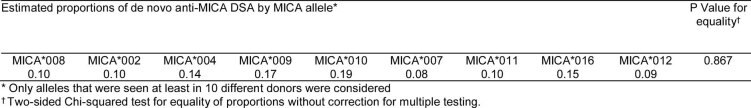
Estimated proportions of de novo anti-MICA DSA depending on donor MICA alleles

**Extended Data Table 6:**
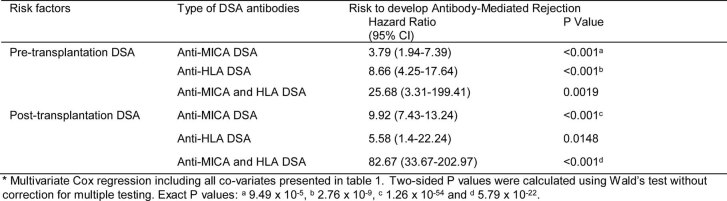
Cumulative impact of anti-MICA and anti-HLA DSA on Antibody-Mediated Rejection*

**Extended Data Table 7:**
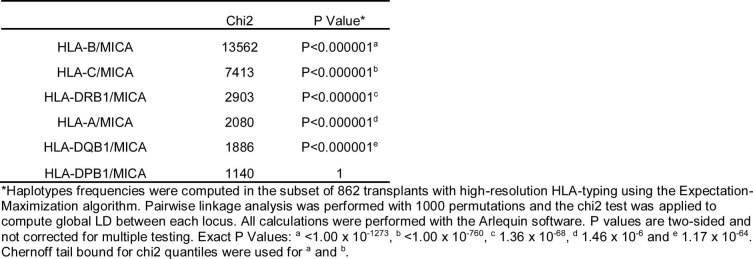
Linkage Disequilibrium between classical *HLA* genes and *MICA*

## Data Availability

All requests for raw or processed data will be promptly reviewed by representatives of all centers having participated in the study, and given that the request is reasonable and complies with the French (and the requestor country’s) national laws and regulations, de-identified data will be shared upon the signing of a data transfer agreement. All such requests should be directly addressed to the corresponding author (S.B.) (siamak@unistra.fr). Source data are provided with this paper.

## Extended data

are available for this paper at 10.1038/s41591-022-01725-2.

## Code availability

The VBA code for *MICA* typing has been deposited and is available at 10.5281/zenodo.5879173 website.
