# Supplementary material for: The MHC class I MICA gene is a histocompatibility antigen in kidney transplantation
Source: Nat Med. 2022 Mar 14;28(5):989–98. doi: 10.1038/s41591-022-01725-2 (PMC9117142; doi:10.1038/s41591-022-01725-2)
Supplement: Supplementary file 1 — Supplementary Figs. 1–4, Supplementary Tables 1–3. [file 41591_2022_1725_MOESM1_ESM.pdf]

nature portfolio

https://doi.org/10.1038/s41591-022-01725-2

Supplementary information

The MHC class I *MICA* gene is a histocompatibility antigen in kidney transplantation

In the format provided by the
authors and unedited

Supplementary Figures

Supplementary Figure 1. Kaplan-Meier curves for kidney graft survival according to *MICA*-eplet matching status. The probability of graft survival is shown for patients with no MICA eplet mismatches versus those with at least one MICA eplet mismatch. Two-sided log-rank test P value without correction is shown.

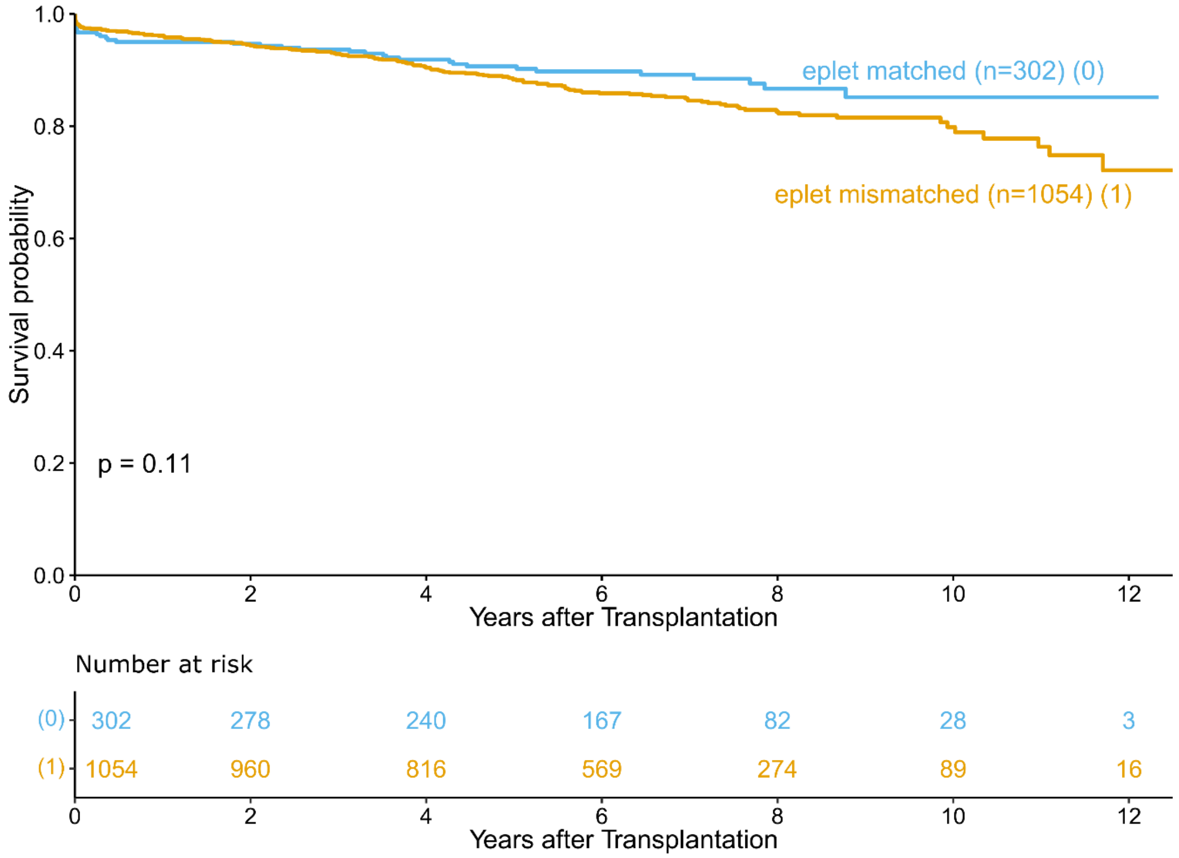

Supplementary Figure 2. Cumulative incidence of antibody-mediated rejection according to the presence or absence of pre-transplantation eplet-specific anti-MICA DSA.

Two-sided log-rank test P value without correction is shown.

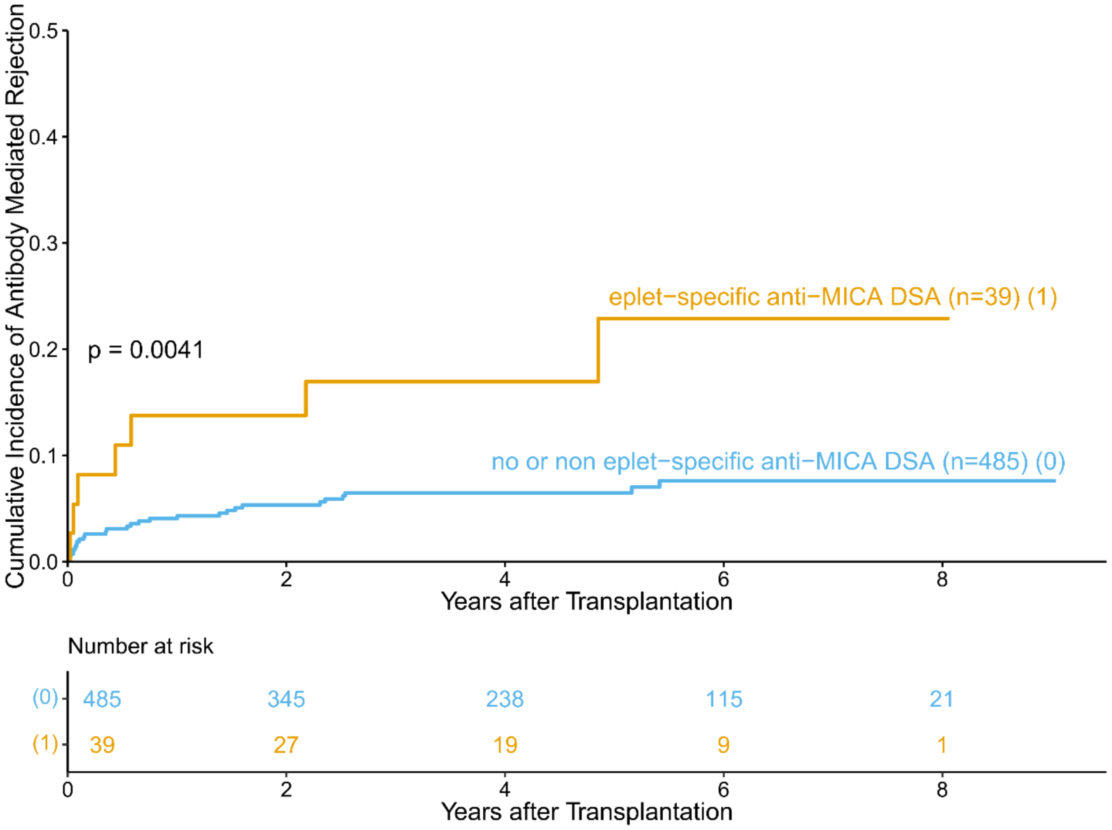

Supplementary Figure 3. Cumulative incidence of antibody-mediated rejection according to the presence or absence of post-transplantation (one year) eplet-specific anti-MICA DSA. Two-sided log-rank test P value without correction is shown.

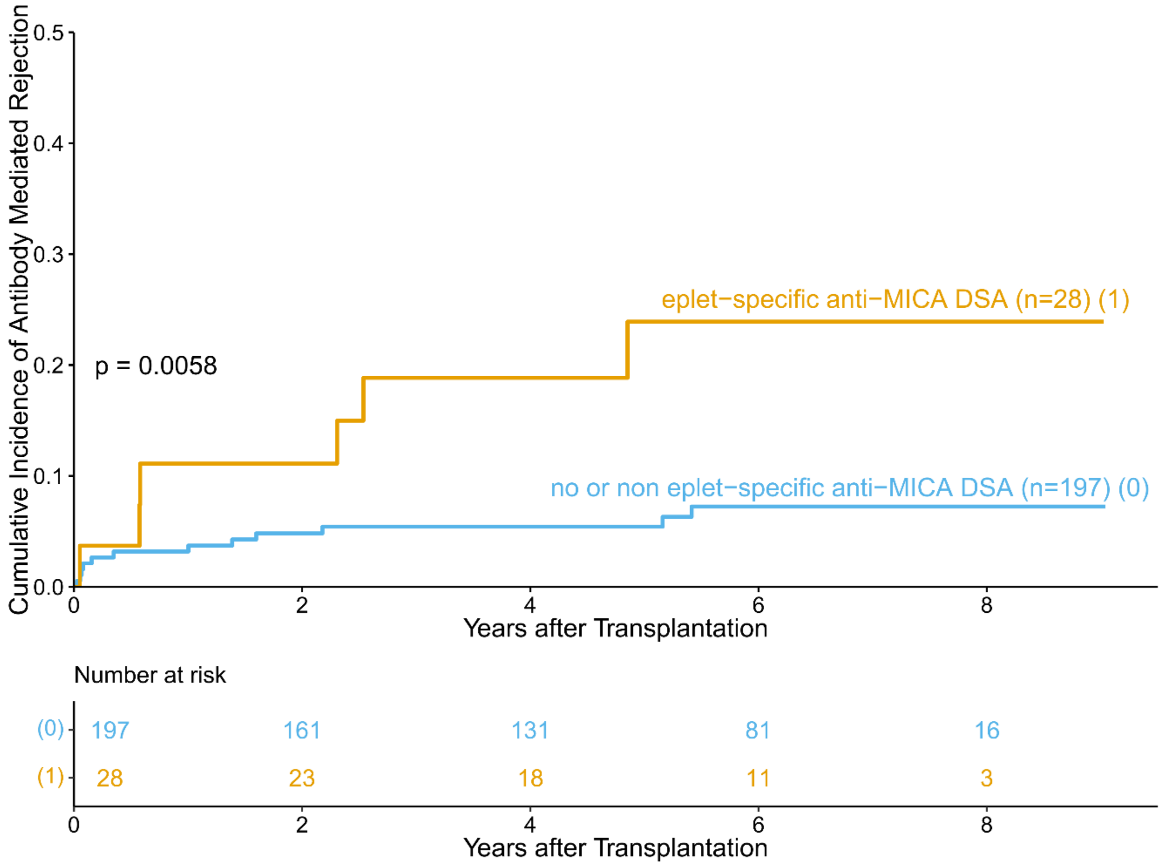

Supplementary Figure 4. ROC analysis representing specificity and sensitivity to detect ABMR with varying MFI values of anti-MICA DSA at one year post-transplantation. A MFI of 100 corresponds to a sensitivity of 62.5% and a specificity of 82.2%.

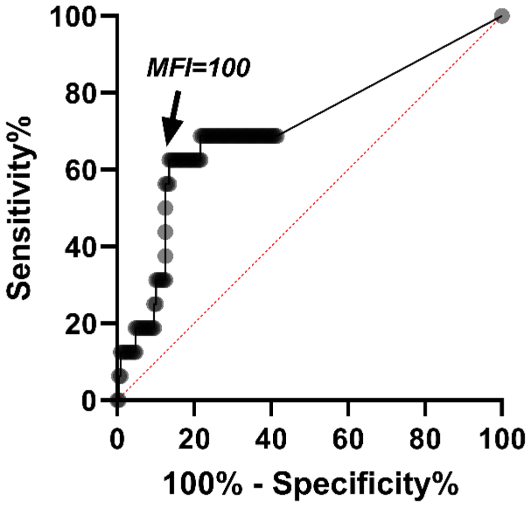

Supplementary Tables

Supplementary Table 1. Demographics of 524 patients characterized for pre-formed anti-MICA and anti-HLA donor-specific antibodies.

	Total transplants (n = 524)	no DSA MICA (n = 447)	DSA MICA (n = 77)	P-value*
Transplantation centers[†]				0.15
Nancy	133 (25.4%)	117 (26.2%)	16 (20.8%)	
Nantes	179 (34.2%)	158 (35.3%)	21 (27.3%)	
Necker	132 (25.2%)	104 (23.3%)	28 (36.4%)	
Saint-Louis	41 (7.8%)	34 (7.6%)	7 (9.1%)	
Toulouse	39 (7.4%)	34 (7.6%)	5 (6.5%)	
Age of donor (years)				0.84
< 43	136 (26%)	114 (25.5%)	22 (28.6%)	
43-64	258 (49.2%)	222 (49.7%)	36 (46.8%)	
65 or older	130 (24.8%)	111 (24.8%)	19 (24.7%)	
Sex of donor				0.75
Female	226 (43.1%)	191 (42.7%)	35 (45.5%)	
Male	298 (56.9%)	256 (57.3%)	42 (54.5%)	
Living/Deceased donor status				0.41
Living	39 (7.4%)	31 (6.9%)	8 (10%)	
Deceased	485 (92.6%)	416 (93.1%)	69 (89.6%)	
Age of recipient (years)				0.24
< 44	138 (26.3%)	118 (26.4%)	20 (26%)	
44-61	261 (49.8%)	217 (48.5%)	44 (57.1%)	
62 or older	125 (23.9%)	112 (25.1%)	13 (16.9%)	
Sex of recipient				0.62
Female	161 (30.7%)	135 (30.2%)	26 (33.8%)	
Male	363 (69.3%)	312 (69.8%)	51 (66.2%)	
BMI of recipient (median)				0.46
<= 24.3	261 (49.8%)	226 (50.6%)	35 (45.5%)	
> 24.3	261 (49.8%)	219 (49%)	42 (54.5%)	
Missing	2 (0.4%)	2 (%0.4)	0 (0%)	
Time from dialysis to transplantation (months)				0.22
<= 30	228 (43.5%)	202 (45.2%)	26 (33.8%)	
> 30	228 (43.5%)	192 (43%)	36 (46.7%)	
Missing	68 (13%)	53 (11.8%)	15 (19.5%)	
End-stage kidney disease [‡]				0.03
Other	494 (94.3%)	426 (95.3%)	68 (88.3%)	
Potential recurrent nephropathy	30 (5.7%)	21 (4.7%)	9 (11.7%)	
Donor-Recipient CMV status				0.07
Negative-Negative	108 (20.6%)	99 (22.1%)	9 (11.7%)	
Negative-Positive	145 (27.7%)	116 (26%)	29 (37.7%)	
Positive-Negative	91 (17.4%)	79 (17.7%)	12 (15.6%)	
Positive-Positive	176 (33.6%)	149 (33.3%)	27 (35.1%)	
Missing	4 (0.8%)	4 (0.9%)	0 (0%)	
Year of transplantation				1
< 2007	99 (18.9%)	84 (18.8%)	15 (19.5%)	
>= 2007	425 (81.1%)	363 (81.2%)	62 (80.5%)	
Cold ischemia time (minutes) (24 hours)				0.6
<= 1440	407 (77.7%)	350 (78.3%)	57 (74%)	
> 1440	115 (21.9%)	96 (21.5%)	19 (24.7%)	
Missing	2 (0.4%)	1 (0.2%)	1 (1.3%)	
HLA-A incompatibilities				0.46
0	94 (17.9%)	83 (18.6%)	11 (14.3%)	
1 or 2	430 (82.1%)	364 (81.4%)	66 (85.7%)	
HLA-B incompatibilities				0.28
0	56 (10.7%)	51 (11.4%)	5 (6.5%)	
1 or 2	468 (89.3%)	396 (88.6%)	72 (93.5%)	
HLA-DR incompatibilities				0.51
0	124 (23.7%)	103 (23%)	21 (27.3%)	
1 or 2	400 (76.3%)	344 (77%)	56 (72.7%)	
DSA HLA pre-transplantation				0.33
No	475 (90.6%)	408 (91.3%)	67 (87%)	
Yes	49 (9.4%)	39 (8.7%)	10 (13%)	
Delayed Graft Function (days)				0.46
0	334 (63.7%)	281 (62.9%)	53 (68.8%)	
1 or more	156 (29.8%)	136 (30.4%)	20 (26%)	
Missing	34 (6.5%)	30 (6.7%)	4 (5.2%)	
Type of graft				0.77
Kidney or Kidney+Kidney	475 (90.6%)	404 (90.4%)	71 (92.2%)	
Kidney + Pancreas	49 (9.4%)	43 (9.6%)	6 (7.8%)	
Graft rank				0.005
First transplant	486 (92.7%)	421 (94.2%)	65 (84.4%)	
Retransplantation	38 (7.3%)	26 (5.8%)	12 (15.6%)	
Induction treatment[§]				0.33
No induction treatment	41 (7.8%)	38 (8.5%)	3 (3.9%)	
Non-depleting induction	281 (53.6%)	240 (53.7%)	41 (53.2%)	
Depleting induction	202 (38.5%)	169 (37.8%)	33 (42.9%)	

Results are presented as number of patients and corresponding percentages of the study population.
HLA: Human Leukocyte Antigen. All clinical variables of the table were used for adjustment in the multivariate models.

* Two-sided P values were determined with the Pearson's Chi square test or the Fisher's exact test and were not corrected for multiple testing.

† Patients received their transplant in six centers member of DIVAT ("**D**onnées **I**nformatisées et **VA**lidées en **T**ransplantation") consortium.

‡ Potential recurrent nephropathy includes: focal segmental glomerulosclerosis, IgA nephropathy, type I and II membranoproliferative glomerulonephritis, membranous glomerulonephritis, Wegener's granulomatosis, systemic lupus erythematosus, scleroderma and hemolytic uremic syndrome.

§ Induction therapy was performed with anti-thymocyte globulin or anti-CD3 antibody (depleting) or anti-IL2 receptor antibody (non-depleting).

Supplementary Table 2. Demographics of 225 Patients characterized at one year post-allograft anti-MICA and anti-HLA donor-specific antibodies

	Total transplants (n = 225)	No DSA MICA (n = 176)	DSA MICA (n = 49)	P Value*
Transplantation centers[†]				0.14
Nancy	3 (1.3%)	2 (1.1%)	1 (2%)	
Nantes	138 (61.3%)	112 (63.6%)	26 (53.1%)	
Necker	66 (29.3%)	46 (26.1%)	20 (40.8%)	
Saint-Louis	18 (8%)	16 (9.1%)	2 (4.1%)	
Age of donor (years)				0.27
< 41	58 (25.8%)	44 (25%)	14 (28.6%)	
41-63	118 (52.4%)	97 (55.1%)	21 (42.9%)	
64 or older	49 (21.8%)	35 (19.9%)	14 (28.6%)	
Sex of donor				0.68
Female	93 (41.3%)	71 (40.3%)	22 (44.9%)	
Male	132 (58.7%)	105 (59.7%)	27 (55.1%)	
Living/Deceased donor status				1
Living	7 (3.1%)	6 (3.4%)	1 (2%)	
Deceased	218 (96.9%)	170 (96.6%)	48 (98%)	
Age of recipient (years)				0.83
< 42	58 (25.8%)	47 (26.7%)	11 (22.4%)	
42-59	115 (51.1%)	89 (50.6%)	26 (53.1%)	
60 or older	52 (23.1%)	40 (22.7%)	12 (24.5%)	
Sex of recipient				1
Female	70 (31.1%)	55 (31.2%)	15 (30.6%)	
Male	155 (68.9%)	121 (68.8%)	34 (69.4%)	
BMI of recipient (median)				0.52
<= 23.2	112 (49.8%)	90 (51.1%)	22 (44.9%)	
> 23.2	112 (49.8%)	85 (48.3%)	27 (55.1%)	
Missing	1 (0.4%)	1 (0.6%)	0 (0%)	
Time from dialysis to transplantation (months)				0.61
<= 32	94 (41.8%)	77 (43.8%)	17 (34.7%)	
> 32	95 (42.2%)	74 (42%)	21 (42.9%)	
Missing	36 (16%)	25 (14.2%)	11 (22.4%)	
End-stage kidney disease [‡]				0.53
Other	210 (93.3%)	163 (92.6%)	47 (95.9%)	
Potential recurrent nephropathy	15 (6.7%)	13 (7.4%)	2 (4.1%)	
Donor-Recipient CMV status				0.93
Negative-Negative	52 (23.1%)	41 (23.3%)	11 (22.4%)	
Negative-Positive	64 (28.4%)	51 (29%)	13 (26.5%)	
Positive-Negative	38 (16.9%)	30 (17%)	8 (16.3%)	
Positive-Positive	69 (30.7%)	52 (29.5%)	17 (34.7%)	
Missing	2 (0.9%)	2 (1.1%)	0 (0%)	
Year of transplantation				0.56
< 2008	148 (65.8%)	118 (67%)	30 (61.2%)	
>= 2008	77 (34.2%)	58 (33%)	19 (38.8%)	
Cold ischemia time (minutes) (24 hours)				0.75
<= 1440	167 (74.2%)	132 (75%)	35 (71.4%)	
> 1440	58 (25.8%)	44 (25%)	14 (28.6%)	
HLA-A incompatibilities				0.81
0	41 (18.2%)	31 (17.6%)	10 (20.4%)	
1 or 2	184 (81.8%)	145 (82.4%)	39 (79.6%)	
HLA-B incompatibilities				0.85
0	27 (12%)	22 (12.5%)	5 (10.2%)	
1 or 2	198 (88%)	154 (87.5%)	44 (89.8%)	
HLA-DR incompatibilities				0.64
0	43 (19.1%)	32 (18.2%)	11 (22.4%)	
1 or 2	182 (80.9%)	144 (81.8%)	38 (77.6%)	
DSA HLA pre-transplantation				0.053
No	202 (89.8%)	163 (92.6%)	39 (79.6%)	
Yes	21 (9.3%)	13 (7.4%)	8 (16.3%)	
Missing	2 (0.9%)	0 (0%)	2 (4.1%)	
Delayed Graft Function (days)				0.46
0	143 (63.5%)	108 (61.4%)	35 (71.4%)	
1 or more	69 (30.7%)	56 (31.8%)	13 (26.5%)	
Missing	13 (5.8%)	12 (6.8%)	1 (2%)	
Type of graft				1
Kidney or Kidney+Kidney	186 (82.7%)	145 (82.4%)	41 (83.7%)	
Kidney + Pancreas	39 (17.3%)	31 (17.6%)	8 (16.3%)	
Graft rank				0.02
First transplant	211 (93.8%)	169 (96%)	42 (85.7%)	
Retransplantation	14 (6.2%)	7 (4%)	7 (14.3%)	
Induction treatment[§]				0.27
No induction treatment	7 (3.1%)	4 (2.3%)	3 (6.1%)	
Non-depleting induction	141 (62.7%)	113 (64.2%)	28 (57.1%)	
Depleting induction	77 (34.2%)	59 (33.5%)	18 (36.7%)	

Results are presented as number of patients and corresponding percentages of the study population.
HLA: Human Leukocyte Antigen. All clinical variables of the table were used for adjustment in the multivariate models.
* Two-sided P values were determined with the Pearson's Chi square test or the Fisher's exact test and were not corrected for multiple testing.

[†] Patients received their transplant in six centers member of DIVAT ("**D**onnées **I**nformatisées et **VA**lidées en **T**ransplantation") consortium.
[‡] Potential recurrent nephropathy includes: focal segmental glomerulosclerosis, IgA nephropathy, type I and II membranoproliferative glomerulonephritis, membranous glomerulonephritis, Wegener's granulomatosis, systemic lupus erythematosus, scleroderma and hemolytic uremic syndrome.
[§] Induction therapy was performed with anti-thymocyte globulin or anti-CD3 antibody (depleting) or anti-IL2 receptor antibody (non-depleting).

Supplementary Table 3. Demographics of 168 patients with ABMR.

	Total transplants (n = 168)	DSA MICA (n = 44)	No DSA MICA (n = 124)	P Value*
Age of donor (years)				0.18
< 43	42 (25.0%)	16 (36.4%)	26 (21%)	
43-64	64 (38.1%)	16 (36.4%)	48 (38.7%)	
65 or older	61 (36.3%)	12 (27.2%)	49 (39.5%)	
Missing	1 (0.6%)	0 (0.0%)	1 (0.8%)	
Sex of donor				0.042
Female	87 (51.8%)	17 (38.6%)	70 (56.5%)	
Male	81 (48.2%)	27 (61.4%)	54 (43.5%)	
Living/Dead donor status				0.025
Living	13 (7.7%)	0 (0.0%)	13 (10.5%)	
Dead	155 (92.3%)	44 (100%)	111 (89.5%)	
Age of recipient on the day of the transplantation (years)				0.017
< 44	49 (29.2%)	20 (45.5%)	29 (23.4%)	
44-61	66 (39.3%)	15 (34.1%)	51 (41.1%)	
62 or older	53 (31.5%)	9 (20.4%)	44 (35.5%)	
Sex of recipient				0.62
Female	71 (42.3%)	20 (45.5%)	51 (41.1%)	
Male	97 (57.7%)	24 (54.5%)	73 (58.9%)	
BMI of recipient (median)				0.012
<= 24.3	75 (44.6%)	28 (63.6%)	47 (37.9%)	
> 24.3	92 (54.8%)	16 (36.4%)	76 (61.3%)	
Missing	1 (0.6%)	0 (0.0%)	1 (0.8%)	
Time from dialysis to transplantation (months)				0.14
<= 30	66 (39.3%)	22 (50%)	44 (35.5%)	
> 30	85 (50.6%)	20 (45.5%)	65 (52.4%)	
Missing	17 (10.1%)	2 (4.5%)	15 (12.1%)	
Potential recurrent nephropathy†				0.39
No	137 (81.5%)	34 (77.3%)	103 (83.1%)	
Yes	31 (18.5%)	10 (22.7%)	21 (16.9%)	
Donor-Recipient CMV status				0.087
Negative-Negative	38 (22.6%)	8 (18.2%)	30 (24.2%)	
Negative-Positive	32 (19%)	5 (11.4%)	27 (21.8%)	
Positive-Negative	31 (18.5%)	6 (13.6%)	25 (20.1%)	
Positive-Positive	66 (39.3%)	25 (56.8%)	41 (33.1%)	
Missing	1 (0.6%)	0 (0.0%)	1 (0.8%)	
Year of transplantation				0.0047
< 2014	80 (47.6%)	29 (65.9%)	51 (41.1%)	
>= 2014	88 (52.4%)	15 (34.1%)	73 (58.9%)	
Cold ischemia time (minutes) (24 hours)				0.55
<= 971	82 (48.8%)	19(43.2%)	63 (50.8%)	
> 971	85 (50.6%)	25 (56.8%)	60 (48.4%)	
Missing	1 (0.6%)	0(0.0%)	1 (0.8%)	
DSA HLA class 1 at the time of biopsy				0.047
No	129 (76.8%)	29 (65.9%)	100 (80.6%)	
Yes	39 (23.2%)	15 (34.1%)	24 (19.4%)	
DSA HLA class 2 at the time of biopsy				0.48
No	115 (68.5%)	32 (72.7%)	83 (66.9%)	
Yes	53 (31.5%)	12 (27.3%)	41 (33.1%)	
All DSA HLA at the time of biopsy				0.33
No	87 (51.8%)	20(45.5%)	67 (54.0%)	
Yes	81 (48.2%)	24 (54.5%)	57 (46.0%)	
Delayed Graft Function (days)				0.5
0	110 (65.5%)	27 (61.4%)	83 (66.9%)	
1 or more	51 (30.3%)	16 (36.3%)	35 (28.3%)	
Missing	7 (4.2%)	1 (2.3%)	6 (4.8%)	
Type of graft				0.55
Kidney or Kidney+Kidney	167 (99.4%)	44 (100%)	123 (99.2%)	
Kidney + Pancreas	1 (0.6%)	0 (0.0%)	1 (0.8%)	
Number of transplantation				
First	132 (78.6%)	32 (72.7%)	100 (80.6%)	
Second or more	36 (21.4%)	12 (27.3%)	24 (19.4%)	
Induction treatment‡				0.27
No	7 (4.2%)	0 (0.0%)	7 (5.6%)	
Non-depleting induction	74 (44%)	17 (38.6%)	57 (46%)	
Depleting induction	86 (51.2%)	27(61.4%)	59 (47.6%)	
Missing	1 (0.6%)	0 (0.0%)	1 (0.8%)	
Age on the day of biopsy (years)				0.16
< 44	42 (25%)	15 (34.1%)	27 (21.8%)	
44-61	63 (37.5%)	17 (38.6%)	46 (37.1%)	
62 or older	63 (37.5%)	12 (27.3%)	51 (41.1%)	
Delay between graft and biopsy (months)				0.002
< 13	87 (51.8%)	14 (31.8%)	73 (58.9%)	
>= 13	81 (48.2%)	30 (68.2%)	51 (41.1%)	
Glomerular filtration rate CKD-EPI (ml/min/1.73m)				0.78
>= 31	87 (51.8%)	22 (50.0%)	65 (52.4%)	
< 31	81 (48.2%)	22 (50.0%)	59 (47.6%)	
Proteinuria (g/d)				0.63
<= 0.2	74 (44%)	21 (47.7%)	53 (42.7%)	

> 0.2	72 (42.9%)	19 (43.2%)	53 (42.7%)	
Missing	22 (13.1%)	4 (9.1%)	18 (14.6%)	
Indication of the biopsy				0.041
Systematic	25 (14.9%)	1 (2.3%)	24 (19.4%)	
Graft dysfunction	79 (47%)	26 (59.1%)	53 (42.7%)	
HLA-DSA	38 (22.6%)	10 (22.7%)	28 (22.6%)	
Other	26 (15.5%)	7 (15.9%)	19 (15.3%)	
Microvascular inflammation				0.86
g+cpt ≥ 2	142 (84.5%)	37 (84.1%)	105 (84.7%)	
g+cpt < 2	17 (10.1%)	4 (9.1%)	13 (10.5%)	
Not applicable§	9 (5.4%)	3 (6.8%)	6 (4.8%)	
Glomerulitis score				0.73
g ≥ 1	146 (86.9%)	37 (84.1%)	109 (87.9%)	
g < 1	14 (8.3%)	4 (9.1%)	10 (8.1%)	
Not applicable	8 (4.8%)	3 (6.8%)	5 (4%)	
Peritubular capillaritis score				0.28
ptc ≥ 1	106 (63.1%)	32 (72.7%)	74 (59.7%)	
ptc < 1	54 (32.1%)	10 (22.7%)	44 (35.5%)	
Not applicable	8 (4.8%)	2 (4.6%)	6 (4.8%)	
Initimal arteritis score				0.11
v ≥ 1	17 (10.1%)	1 (2.3%)	16 (12.9%)	
v < 1	142 (84.5%)	40 (90.9%)	102 (82.3%)	
Not applicable	8 (5.4%)	3 (6.8%)	6 (4.8%)	
Transplant glomerulopathy score				0.69
cg ≥ 1	40 (23.8%)	12 (27.3%)	28 (22.6%)	
cg < 1	119 (70.8%)	29 (65.9%)	90 (72.6%)	
Not applicable	9 (5.4%)	3 (6.8%)	6 (4.8%)	
Peritubular C4d staining				0.36
Positive	41 (24.4%)	13 (29.5%)	28 (22.6%)	
Negative	127 (75.6%)	31 (70.5%)	96 (77.4%)	
Thrombotic Microangiopathy				0.78
Positive	9 (5.4%)	2 (4.5%)	7 (5.6%)	
Negative	159 (94.6%)	42 (95.5%)	117 (94.4%)	
Tubulitis score				0.2
i ≥ 1	78 (46.4%)	20 (45.5%)	58 (46.8%)	
t < 1	80 (47.6%)	19 (43.2%)	61 (49.2%)	
Not applicable	10 (6.0%)	5 (11.3%)	5 (4%)	
Interstitial inflammation score				0.29
i ≥ 1	104 (61.9%)	27 (61.3%)	77 (62.1%)	
t < 1	53 (31.6%)	12 (27.3%)	41 (33.1%)	
Not applicable	11 (6.5%)	5 (11.4%)	6 (4.8%)	
T-cell mediated rejection				0.83
i ≥ 2 and t ≥ 2	48 (28.6%)	11 (25.0%)	37 (29.8%)	
i < 2 or t < 2	113 (67.3%)	31 (70.5%)	82 (66.1%)	
Missing	7 (4.1%)	2 (4.5%)	5 (4.1%)	

Results are presented as number of patients and corresponding percentages of the study population.
HLA: Human Leukocyte Antigen. All clinical variables of the table were used for adjustment in the multivariate models.
* Two-sided P values were determined with the Pearson’s Chi square test or the Fisher’s exact test and were not corrected for multiple testing.
† Potential recurrent nephropathy includes: focal segmental glomerulosclerosis, IgA nephropathy, type I and II membranoproliferative glomerulonephritis, membranous glomerulonephritis, Wegener's granulomatosis, systemic lupus erythematosus, scleroderma and hemolytic uremic syndrome.
‡ Induction therapy was performed with anti-thymocyte globulin or anti-CD3 antibody (depleting) or anti-IL2 receptor antibody (non-depleting).
§ Number of non-sclerosed glomeruli inadequate (<=7) for numeric coding.
