# Supplementary material for: The MHC class I MICA gene is a histocompatibility antigen in kidney transplantation
Source: Nat Med. 2022 Mar 14;28(5):989–98. doi: 10.1038/s41591-022-01725-2 (PMC9117142; doi:10.1038/s41591-022-01725-2)
Supplement: Supplementary file 2 — Reporting Summary [file 41591_2022_1725_MOESM2_ESM.pdf]

Corresponding author(s): Seiamak Bahram

Last updated by author(s): Jan 18, 2022

Reporting Summary

Nature Portfolio wishes to improve the reproducibility of the work that we publish. This form provides structure for consistency and transparency in reporting. For further information on Nature Portfolio policies, see our Editorial Policies and the Editorial Policy Checklist.

Statistics

For all statistical analyses, confirm that the following items are present in the figure legend, table legend, main text, or Methods section.

n/a	Confirmed	
☐	☒	The exact sample size (*n*) for each experimental group/condition, given as a discrete number and unit of measurement
☒	☐	A statement on whether measurements were taken from distinct samples or whether the same sample was measured repeatedly
☐	☒	The statistical test(s) used AND whether they are one- or two-sided<br>*Only common tests should be described solely by name; describe more complex techniques in the Methods section.*
☐	☒	A description of all covariates tested
☐	☒	A description of any assumptions or corrections, such as tests of normality and adjustment for multiple comparisons
☐	☒	A full description of the statistical parameters including central tendency (e.g. means) or other basic estimates (e.g. regression coefficient) AND variation (e.g. standard deviation) or associated estimates of uncertainty (e.g. confidence intervals)
☐	☒	For null hypothesis testing, the test statistic (e.g. *F*, *t*, *r*) with confidence intervals, effect sizes, degrees of freedom and *P* value noted<br>*Give P values as exact values whenever suitable.*
☒	☐	For Bayesian analysis, information on the choice of priors and Markov chain Monte Carlo settings
☒	☐	For hierarchical and complex designs, identification of the appropriate level for tests and full reporting of outcomes
☒	☐	Estimates of effect sizes (e.g. Cohen's *d*, Pearson's *r*), indicating how they were calculated

Our web collection on statistics for biologists contains articles on many of the points above.

Software and code

Policy information about availability of computer code

Data collection	The data were retrieved from the clinical database (see Methods for details) into Microsoft Excel 2016. No other softwares were used for data collection.
Data analysis	The statistical analyses were performed using the computer environment R (version 4.0.2) with the CRAN survival package (https://cran.rproject.org/web/packages/survival/index.html). The SeqScape v2.6 and GeneMapper v4.0 softwares (both from ThermoFisher Scientific, Waltham, Massachusetts, USA) were used for sequence and fragment size analyses, respectively. Microsoft Excel 2016 (Microsoft corporation, Redmond, Washington, US) and a VBA code - deposited at https://doi.org/10.5281/zenodo.5879173 - was used for MICA genotyping.

For manuscripts utilizing custom algorithms or software that are central to the research but not yet described in published literature, software must be made available to editors and reviewers. We strongly encourage code deposition in a community repository (e.g. GitHub). See the Nature Portfolio guidelines for submitting code & software for further information.

Data

Policy information about availability of data

All manuscripts must include a data availability statement. This statement should provide the following information, where applicable:

- Accession codes, unique identifiers, or web links for publicly available datasets
- A description of any restrictions on data availability
- For clinical datasets or third party data, please ensure that the statement adheres to our policy

All requests for raw or processed data will be promptly reviewed by representatives of all centers having participated in the study, and given that the request is reasonable and complies with the French (and the requestor country's) national laws and regulations, de-identified data will be shared upon the signing of a data

transfer agreement. All such requests should be directly addressed to the corresponding author (S.B) (siamak@unistra.fr). The VBA code for MICA genotyping has been deposited and is available at https://doi.org/10.5281/zenodo.5879173 website.

Field-specific reporting

Please select the one below that is the best fit for your research. If you are not sure, read the appropriate sections before making your selection.

☒ Life sciences ☐ Behavioural & social sciences ☐ Ecological, evolutionary & environmental sciences

For a reference copy of the document with all sections, see nature.com/documents/nr-reporting-summary-flat.pdf

Life sciences study design

All studies must disclose on these points even when the disclosure is negative.

Sample size	No statistical method was used to predetermine the sample size. All samples from the participating clinical centers that participated in the study and were in conformity with study goals were included in the study. The cohort of 1356 patients corresponds therefore to all ABO-compatible, cross-match negative transplants for which DNA of both the donor and the recipient, as well as a full set of clinical information were available from the participating centers. This constitutes the largest sample size for studying MICA in transplantation and therefore has no precedent. The fact that similar (or inferior) size cohorts have been successfully used in the HLA field and that we did encounter a sufficient number of events within the studied time period which led to statistically significant results, proves that the size of the cohort was sufficient.
Data exclusions	No data was excluded.
Replication	An independent cohort of 168 transplants was used to confirm our findings. This was the sole attempt for replication and it was successful.
Randomization	This is not a clinical trial. It is a retrospective study and therefore randomization is not relevant nor is it applicable.
Blinding	This is not a clinical trial. It is a retrospective study and therefore blinding is not relevant nor is it applicable.

Reporting for specific materials, systems and methods

We require information from authors about some types of materials, experimental systems and methods used in many studies. Here, indicate whether each material, system or method listed is relevant to your study. If you are not sure if a list item applies to your research, read the appropriate section before selecting a response.

Materials & experimental systems

n/a	Involved in the study	
☒	☐	Antibodies
☒	☐	Eukaryotic cell lines
☒	☐	Palaeontology and archaeology
☒	☐	Animals and other organisms
☐	☒	Human research participants
☒	☐	Clinical data
☒	☐	Dual use research of concern

Methods

n/a	Involved in the study	
☒	☐	ChIP-seq
☒	☐	Flow cytometry
☒	☐	MRI-based neuroimaging

Human research participants

Policy information about studies involving human research participants

Population characteristics	The study population consisted of 1356 KTR (and donors) who underwent kidney transplantation between 2002 and 2011. Patients were followed until January 1st, 2015. The transplantation allocation rules were the same for all six centers and followed the recommendations of the French national agency for organ procurement (Agence de la biomédecine, Paris, France). 68% of patients were male and 32% female. The age distribution was the following: <42: 26%; 42-61: 51% and >61: 23%.<br>An independent cohort consisting of 168 patients from Strasbourg University Hospital with a biopsy proven acute ABMR episode that occurred between 2013 and 2018 was also analyzed. These patients had ABMR specific lesions with (n=81) or without anti-HLA DSA (n=87). 58% of patients were male and 42% female. The age distribution was the following: <44: 29%; 44-61: 39% and >61: 32%.
Recruitment	All ABO compatible, cross-match negative transplants for which DNA of both the donor and the recipient, as well as relevant clinical data were available in the participating centers were included, without further selection criteria. No self-selection or other types of biases were present.
Ethics oversight	This retrospective histocompatibility study aimed to examine whether D/R matching at the MICA locus improves the outcomes of kidney transplantations. Kidney transplant recipients (KTRs) (and their donors) from seven French centers were

enrolled. Genomic DNA and sera were collected in each participating center within the course of routine medical care and histocompatibility geno/serotypings. The study was approved by the institutional review boards (IRB) of Nantes University Hospital (CPP Grand Ouest DC-2011-1399, on behalf of all participating centers, except Strasbourg) and Strasbourg University Hospital (CPP Est number DC-2013-1990). The study was performed according to the principles of the Helsinki declaration. Written informed consent was obtained from all participants of both the initial and the independent cohorts.

Note that full information on the approval of the study protocol must also be provided in the manuscript.
